# Microsatellite marker development in the crop wild relative *Linum bienne* using genome skimming

**DOI:** 10.1002/aps3.11349

**Published:** 2020-05-26

**Authors:** Beatrice Landoni, Juan Viruel, Rocio Gómez, Robin G. Allaby, Adrian C. Brennan, F. Xavier Picó, Rocio Pérez‐Barrales

**Affiliations:** ^1^ School of Biological Sciences University of Portsmouth PO1 2DY Portsmouth United Kingdom; ^2^ Royal Botanic Gardens, Kew TW9 3AE Richmond, London United Kingdom; ^3^ Departamento de Ecología Integrativa Estación Biológica de Doñana (EBD) Consejo Superior de Investigaciones Científicas (CSIC) 41092 Seville Spain; ^4^ School of Life Sciences University of Warwick CV4 7AL Warwick United Kingdom; ^5^ Department of Biosciences Durham University Stockton Road DH1 3LE Durham United Kingdom

**Keywords:** crop wild relative, Linaceae, *Linum bienne*, pale flax, population genetics, simple sequence repeat (SSR)

## Abstract

**Premise:**

Nuclear microsatellite markers were developed for *Linum bienne*, the sister species of the crop *L. usitatissimum*, to provide molecular genetic tools for the investigation of *L. bienne* genetic diversity and structure.

**Methods and Results:**

Fifty microsatellite loci were identified in *L. bienne* by means of genome skimming, and 44 loci successfully amplified. Of these, 16 loci evenly spread across the *L. usitatissimum* reference nuclear genome were used for genotyping six *L. bienne* populations. Excluding one monomorphic locus, the number of alleles per locus ranged from two to 12. Four out of six populations harbored private alleles. The levels of expected and observed heterozygosity were 0.076 to 0.667 and 0.000 to 1.000, respectively. All 16 loci successfully cross‐amplified in *L. usitatissimum*.

**Conclusions:**

The 16 microsatellite loci developed here can be used for population genetic studies in *L. bienne*, and 28 additional loci that successfully amplified are available for further testing.

The genus *Linum* L. (Linaceae) includes 180–200 species, with most species diversity concentrated in the Mediterranean Basin. It has become an important plant group to investigate the evolution of breeding systems and genome duplication events (Sveinsson et al., 2014; Ruiz‐Martín et al., 2018). *Linum* includes *L. usitatissimum* L., cultivated globally for fiber and oil, and its wild relative *L. bienne* Mill. (Fu, 2019). The two share a whole‐genome duplication that occurred 5–9 mya (Sveinsson et al., 2014). Although phenotypic and genotypic variation of flax have been studied in relation to crop improvement (Fu, 2019), population variation in *L. bienne* remains relatively unexplored (but see Uysal et al., 2012).

*Linum usitatissimum* is an annual species, whereas *L. bienne* is a winter annual or perennial, growing in isolated patches across the Middle East, the Mediterranean Basin, and Western Europe (Uysal et al., 2012). For both species, seed production relies on self‐pollination and, while outcrossing is rare, it has been central to the adaptation of the crop to northern latitudes by means of gene flow from *L. bienne* to *L. usitatissimum* (Gutaker et al., 2019). Sertse et al. (2019) highlighted the importance of eco‐geographic factors in shaping *L. usitatissimum* genetic structure, and noted that the Mediterranean region is poorly represented in its core collection. Interestingly, the geographic distribution of *L. bienne* spans this area. Additionally, genotypic and phenotypic characterization of Turkish *L. bienne* populations has identified patterns of local adaptation (Uysal et al., 2010, 2012). Taken together, these studies reveal the potential value of *L. bienne* for crop improvement and evolutionary research. Most of the molecular tools available for *L. bienne*, including microsatellite markers, were retrieved ad hoc from those developed in *L. usitatissimum*, and population‐level variation was not explored (Cloutier et al., 2012; Soto‐Cerda et al., 2014). Only Uysal et al. (2010) genotyped *L. bienne* populations with inter‐simple sequence repeat (ISSR) markers, but from a limited geographical range. Here, we screen 50 microsatellite markers that will serve to investigate genetic diversity and structure of *L. bienne*.

## METHODS AND RESULTS

To identify microsatellite markers, we employed the approach used by Viruel et al. (2018), in which contigs are mined for microsatellite loci after a de novo assembly. DNA extractions for seven *L. bienne* individuals from different locations (Appendix 1) and corresponding whole genome shotgun libraries were prepared following the methods in Viruel et al. (2019). Equimolar pooled libraries (150 × 150 bp) were sequenced at Novogene (Beijing, China) in an Illumina HiSeq X lane (Illumina, San Diego, California, USA). Contigs generated by assembling raw reads with SPAdes version 3.13 (Bankevich et al., 2012) were mapped against a *L. usitatissimum* nuclear genome reference (GenBank IDs CP027619.1–CP027633.1) in BWA version 0.7.17 (Li and Durbin, 2009). The mapping contigs were then scanned for di‐, tri‐, and tetranucleotide repeat motifs with MSATCOMMANDER version 1.0.8 (Faircloth, 2008) using default settings to design primers. Contigs containing microsatellite loci were filtered in R version 3.5.2 (R Core Team, 2018) using a custom‐made script. Loci with primers that met the following requirements were retained: pair penalty <1.7, left‐right penalty <0.8, difference in melting temperature <2°C, primer distance from locus >20 bp, and pair product size between 89 and 301 bp. Polymorphic loci were then identified by BLASTing all contigs mapping to the *L. usitatissimum* reference genome for seven *L. bienne* individuals against the filtered contigs containing microsatellite loci, using BLAST version 2.2.31 (Altschul et al., 1990). Finally, 50 loci (Appendix 2) were left after filtering in R version 3.5.2 (R Core Team, 2018) based on BLAST output. Only microsatellite loci with the following features were retained: ≥4 repeats of the base motif, <5 mismatches between BLAST match and reference, and at least one individual per BLAST group differed from the reference in number of motif repeats. The code used for de novo assembly and selection of microsatellite loci is available in Appendix S1.

For in vivo testing, DNA was extracted from seedlings of six *L. bienne* populations as well as other *Linum* species (Appendix 1). DNA extractions were performed with the ISOLATE II Plant DNA kit (Bioline, London, United Kingdom), using approximately 20 mg of dry leaf material and following the kit protocol with buffer PA1. The 50 loci were first amplified in seven individuals following the *Taq* DNA Polymerase Master Mix instructions (ThermoFisher Scientific, Waltham, Massachusetts, USA). The PCR program consisted of an initial denaturation of 2 min at 94°C; 35 cycles of 1 min at 94°C, 1 min at 56°C (annealing temperature [*T*_a_]), and 2 min at 72°C; and a final extension step of 10 min at 72°C. For 12 out of 50 primer pairs, these conditions did not lead to amplification or produced multiple bands. When multiple bands were obtained, we tested the primers again by increasing *T*_a_ by 1°C. In situations where no initial amplification occurred, we decreased *T*_a_ by 1°C. In total, 44 loci amplified successfully at the end of this process (Appendix 2), with sizes as expected from MSATCOMMANDER output. To genotype all individuals, 16 loci were selected (Table 1) based on maximizing dispersion along the genome, the visual identification of polymorphisms on agarose gels, and avoiding the overlap of peaks during capillary electrophoresis by varying the PCR product sizes. PCR products were pooled in mixes of four loci, and reverse primers were tagged with four different fluorochromes (Table 1). PCR products were electrophoresed on an ABI PRISM 3700 DNA analyzer (Applied Biosystems, Foster City, California, USA), along with a GeneScan 500 LIZ fluorescent internal size standard. Transferability was also tested in three additional *Linum* species, including *L. usitatissimum* (Appendix 1), for the subset of 16 loci.

**Table 1:** Characteristics of 17 microsatellite loci developed for *Linum bienne* via genome skimming using the *L. usitatissimum* genome as a reference to identify a putative chromosome for each locus.

Chromosome	Locus^a^	Primer sequences (5’‐3’)	Repeat motif	Allele size range (bp)	Mix^b^	Fluorescent dye^c^	GenBank accession no.
chr1	ssr1.4	F: CGAGCTCCGTTATCTCCGAG	(AGC)_5_	127–136	4	PET	MN450483
		R: ACGAATCTGAAATGGCGCTG					
chr2	ssr2.1	F: AAAGAAATGCAGAGCGGGAG	(AGG)_4_	215–233	1	PET	MN450485
		R: GCGTCATTTACTCAGTGGCC					
chr2	ssr2a.2	F: CCGTTGCTCTTCCACCAAAG	(AG)_5_	280–282	2	PET	MN450486
		R: CATCTTCACCGTTCAGCTCG					
chr2	ssr2b.2	F: CCGTTGCTCTTCCACCAAAG	(AG)_5_	331–337	2	PET	MN450486
		R: CATCTTCACCGTTCAGCTCG					
chr3	ssr3.2	F: GTCTGCATTGCGATCAGAGG	(AT)_8_	153–163	2	VIC	MN450489
		R: GATAGGTGCCTTGTTCTGCG					
chr3	ssr3.4	F: CAGATTCAACCGTTGCTCCC	(AT)_8_	226–252	4	VIC	MN450487
		R: TTGCCTGTTTCCAACGAGAC					
chr4	ssr4.2	F: TCGTCCTTGATCCTTCCAGC	(ATC)_5_	200–206	2	NED	MN450493
		R: AAGACCCTCAACTCCAACCC					
chr4	ssr4.3	F: ATAGCTGCCAACTTGACTGC	(AAG)_5_	127–130	3	PET	MN450492
		R: TTTCCTAGGACCAGCGACTG					
chr6	ssr6.1	F: TTACACGAGGGATTGCAAGC	(AG)_6_	157–163	1	VIC	MN450500
		R: ACTAGTGAGTCTGCAGTGCC					
chr9	ssr9.3	F: TACGCCAAACACAAGCATCC	(AC)_4_	185–187	3	VIC	MN450514
		R: CAACCCAACCATACCAACCG					
chr10	ssr10.1	F: TCTACAATGGCGACTCAGGG	(AG)_5_	119–127	1	NED	MN450518
		R: CGAATCGGTCAGCGGAATTG					
chr11	ssr11.1	F: CTTCATCTCCGCTTGTTCCG	(AAC)_5_	187–193	1	FAM	MN450519
		R: CATTGGCTGGGCAAGTATGG					
chr11	ssr11.2	F: TGTGCGCAATATGGGTTACG	(AAC)_4_	243–264	2	FAM	MN450520
		R: ACCCACCATCCTTTCTCCAC					
chr11	ssr11.4	F: AAACCAACATCCCACTTGCG	(AG)_4_	292–298	4	NED	MN450521
		R: TTCCAACTGAAAGACGCTCG					
chr12	ssr12.3	F: GGCCACGAATTCCCTCATTC	(AAG)_5_	219–225	3	NED	MN450523
		R: TGGGAAGAACAGTACGGTCC					
chr12	ssr12.4	F: CTACCCTTCTCAGCTCTGCC	(AG)_5_	174–194	4	FAM	MN450522
		R: TTGTGTGCACTTCAAAGCCC					
chr14	ssr14.3^d^	F: ACATTCGCAACTGTATCGCC	(ACT)_4_	280	3	FAM	MN450527
		R: GCGTTTAGGTGGTGGAAAGG					

^a^For all primer pairs, the annealing temperature was 56°C.

a ^b^Loci were pooled into four groups (mixes 1 to 4) for capillary electrophoresis.

b ^c^For each capillary electrophoresis mix containing four loci, four different dyes (PET, VIC, NED, FAM) were used to tag the reverse primer of each pair to facilitate genotyping.

c ^d^Locus 14.3 was monomorphic across all populations, so genetic diversity parameters were not computed for this locus.

Genotyping was conducted manually in Peak Scanner Software version 1.0 (Applied Biosystems). Genetic diversity analyses are presented in Table 2. Allele number and observed heterozygosity (*H*_o_) were estimated with the R package hierfstat version 0.04‐22 (Goudet, 2005). Unbiased expected heterozygosity (*H*_s_), departure from Hardy–Weinberg equilibrium (HWE), linkage disequilibrium, and number of private alleles were calculated using the R package poppr version 2.8.3 (Kamvar et al., 2014).

**Table 2:** Genetic diversity parameters of 16 polymorphic microsatellite loci in six populations of *Linum bienne* a and across all populations.

Locus	All	11 (*n* = 23)				6 (*n* = 23)				IOW2 (*n* = 24)				LLA (*n* = 24)				SUT (*n* = 30)				VIL (*n* = 29)			
	*A*	*A*	*A*_p_	*H*o	*H*s	*A*	*A*_p_	*H*o	*H*s	*A*	*A*_p_	*H*o	*H*s	*A*	*A*_p_	*H*o	*H*s	*A*	*A*_p_	*H*o	*H*s	*A*	*A*_p_	*H*o	*H*s
ssr1.4	4	3	1	1.000	0.627	2	0	0.957	0.510	3	0	1.000	0.550	3	0	0.962	0.563	3	0	1.000	0.554	2	0	1.000	0.508
ssr2.1	5	2	0	0.000	0.464	3	1	0.000	0.456	1	0	0.000^b^	0.000	2	1	0.000	0.444	3	1	0.000	0.129	1	0	0.000^b^	0.000
ssr2a.2	3	1	0	0.000^b^	0.000	1	0	0.000^b^	0.000	2	0	0.458^b^	0.403	3	1	0.731^b^	0.514	2	0	0.033	0.259	2	0	0.000	0.495
ssr2b.2	4	2	0	1.000	0.511	2	0	0.913	0.507	2	0	0.000	0.511	2	0	0.000	0.362	2	1	0.000	0.066	1	0	0.000^b^	0.000
ssr3.2	4	3	0	0.000	0.510	2	0	0.000	0.085	1	0	0.000^b^	0.000	3	0	0.000	0.278	4	0	0.000	0.190	2	0	0.000	0.063
ssr3.4	12	2	0	0.000	0.474	6	3	0.000	0.677	2	0	1.000	0.511	4	1	0.609	0.571	7	3	0.900	0.629	3	0	0.969	0.522
ssr4.2	3	2	0	0.000	0.394	2	0	0.000	0.502	2	0	0.000	0.394	2	0	0.000	0.265	2	0	0.000	0.127	1	0	0.000^b^	0.000
ssr4.3	2	1	0	0.000^b^	0.000	1	0	0.000^b^	0.000	1	0	0.000^b^	0.000	2	0	0.000	0.265	2	0	0.000	0.183	1	0	0.000^b^	0.000
ssr6.1	4	2	0	0.000	0.394	3	0	0.000	0.676	1	0	0.000^b^	0.000	2	0	0.000	0.274	4	1	0.000	0.190	2	0	0.000	0.063
ssr9.3	2	2	0	0.000	0.085	2	0	0.000	0.162	2	0	0.000	0.082	2	0	0.000	0.265	1	0	0.000	0.000	2	0	0.000	0.119
ssr10.1	5	2	0	1.000	0.511	4	1	1.000	0.731	2	0	1.000	0.511	3	0	1.000	0.595	4	0	1.000	0.555	3	0	1.000	0.524
ssr11.1	2	2	0	1.000	0.511	2	0	0.609	0.433	2	0	1.000	0.511	2	0	1.000	0.511	2	0	0.967	0.508	2	0	1.000	0.508
ssr11.2	6	3	0	1.000	0.610	3	0	1.000	0.572	2	0	1.000	0.511	4	0	1.000	0.598	6	0	1.000	0.602	4	0	1.000	0.554
ssr11.4	4	2	0	0.000	0.085	2	0	0.000	0.162	3	0	0.750	0.551	3	0	0.231	0.363	3	1	0.000	0.445	2	0	0.032^b^	0.032
ssr12.3	3	1	0	0.000^b^	0.000	1	0	0.000^b^	0.000	2	0	0.625	0.439	2	0	0.115^b^	0.111	3	1	0.900	0.518	2	0	0.969	0.507
ssr12.4	7	4	0	1.000	0.762	4	0	1.000	0.762	4	0	1.000	0.621	6	1	1.000	0.773	4	0	1.000^b^	0.555	4	0	1.000	0.566
Mean	4.375	2.125	—	0.375	0.371	2.500	—	0.342	0.390	2.000	‐	0.490	0.350	2.813	—	0.416	0.422	3.250	‐	0.425	0.344	2.125	‐	0.436	0.279
SD	2.395	0.781	—	0.484	0.244	1.275	—	0.450	0.268	0.791	‐	0.456	0.232	1.073	—	0.443	0.170	1.521	‐	0.479	0.212	0.927	‐	0.490	0.246

Note

*A* = number of alleles per locus; *A*_p_ = number of private alleles; *H*_o_ = observed heterozygosity; *H*_s_ = unbiased expected heterozygosity; *n* = number of individuals sampled.

a Voucher and locality information are provided in Appendix 1.

b In Hardy–Weinberg equilibrium (*P* > 0.05).

All 16 loci selected for genotyping amplified in *L. bienne* (1.23% of missing data on average), but cross‐amplification was successful only in *L. usitatissimum*. In *L. bienne*, locus ssr14.3 was monomorphic and therefore excluded from the analyses. Locus ssr2.2 included two different microsatellite regions that were then treated as independent loci (ssr2a.2 and ssr2b.2). The number of alleles per locus varied between two and 12 over all six *L. bienne* populations. All populations harbored one to three private alleles for one or more loci, except for populations VIL and IOW2. Depending on the population, 12 to 16 loci significantly deviated from HWE (*P* < 0.05). When loci were in HWE, it was mostly due to fixed alleles (Table 2). *H*_o_ ranged between 0.000 and 1.000, and *H*_S_ ranged between 0.000 and 0.773, across populations and loci. Linkage disequilibrium fluctuated between −0.336 and 1.000, with varying percentages of loci pairs in linkage disequilibrium within populations (between 9% and 54%, *P* < 0.05) (Appendix S2).

The high *H*_o_ and frequent deviation from HWE (Table 2) might arise from fixed alleles on different paralogs produced by past polyploidization events in the genus *Linum*, which was also observed by Cloutier et al. (2012). If duplication is assumed when genotyping, consistency is essential while scoring loci showing a heterozygote fingerprint. Whether the latter is considered the result of homozygosity, heterozygosity, or a combination of both at the duplicated locus will affect estimates of allele frequencies.

## CONCLUSIONS

Microsatellite loci are ideal for providing fine‐scale geographic and temporal information about population genetic processes such as relatedness. The set of loci developed here are distributed across the genome and will therefore be useful to distinguish between genome‐wide processes caused by demography and locus‐specific processes such as adaptation. However, putative paralogy needs investigation. The sequencing of different alleles and additional analysis of the genomic data set could serve to discriminate between paralog copies.

## AUTHOR CONTRIBUTIONS

R.P.B., A.C.B., B.L., and R.G.A. collected the plant material; R.G., B.L., and J.V. conducted the lab work; B.L. and J.V. implemented the genome skimming pipeline; B.L. analyzed the data and wrote the manuscript; R.P.B. and F.X.P. provided the funding and coordinated the work; all authors contributed to reviewing the manuscript.

## Supporting information


**APPENDIX S1.** Script for de novo genome assembly using six *Linum bienne* individuals of different geographical origin, subsequent microsatellite loci mining and primer design, and in silico genotyping.

**APPENDIX S2.** Index of association for 16 polymorphic loci included in this study.

## Data Availability

Raw reads used for genome skimming were deposited in the National Center for Biotechnology Information (NCBI) Sequence Read Archive (BioProject ID: PRJNA580472). Sequence information for microsatellite loci was deposited in NCBI's GenBank, and accession numbers are provided in Table 1 and Appendix 2.

## APPENDIX 1 Voucher information for populations of *Linum bienne*,* L. usitatissimum*, and other *Linum* species used in this study.

**Table:**

Species	Population^a^	*n*	Locality	Latitude	Longitude	Altitude (m)	Voucher accession no.^b^	Sample^c^
*L. bienne* Mill.	**6**	23	Constantina‐Cazalla de la Sierra, Seville, Spain	37.93551111	−5.711172222	529	202011	—
*L. bienne*	**11**	23	La Aliseda, Finca La Inmmediata (Km 3), Jaén, Spain	38.33105278	−3.580855556	710	202012	L17
*L. bienne*	**IOW2**	24	Bembridge, Isle of Wight, United Kingdom	50.68183333	−1.074916667	9	202013	—
*L. bienne*	**LLA**	24	Llanes, Asturias, Spain	43.407375	−4.687527778	26	202014	L58
*L. bienne*	**SUT**	30	Sutton, Nottinghamshire, United Kingdom	53.35291111	−0.959269444	15	202015	—
*L. bienne*	**VIL**	29	Villeneuve, Charente Maritime, France	45.09393056	−1.050338889	21	202016	L49
*L. bienne*	L01	1	Pierrefeu‐du‐Var, Provence‐Alpes‐Côte d'Azur, France	43.25533	6.23802	200	202017	L01
*L. bienne*	CGA1	1	Capo Gallo, Palermo, Sicily, Italy	38.2165	13.32183333	53	202018	L68
*L. bienne*	TYM	1	Ty Mawr Holiday Park, Debinghshire, United Kingdom	53.30307222	−3.553280556	5	202019	L46
*L. bienne*	W77	1	Greece	40.0875	21.722222	835	Collection Gutaker et al. (2019)	L80
*L. usitatissimum* L.	Cultivars Aramis and Volga	2	Terre de Lin, Saint‐Pierre‐Le‐Viger, France	46.227638	2.213749	100	2020110	—
*L. usitatissimum*	Cultivar Gisa and Primus	2	Italy	41.87194	12.56738	—	260080 and 247707	—
*L. usitatissimum*	Cultivar Raba0189	1	Morocco	31.791702	−7.09262	—	247713	—
*L. suffruticosum* L.	—	6	Puerto de las Palomas, Sierra de Grazalema, Cádiz, Spain	36.80	−5.41	400	1449143 and 1054224	—
*L. tenue* Desf.	—	9	El Castillejo Botanical Garden, El Bosque, Cádiz, Spain	36.765210	−5.498114	298	Live collection	—

^a^ *Linum bienne* populations used for genotyping in vivo are in bold.

^b^ For populations 6, 11, IOW2, LLA, VIL, CGA1, L01, and TYM, vouchers were deposited in Portsmouth Natural History Museum (PORMG, Portsmouth, United Kingdom); for *L. usitatissimum*, the registered cultivars Aramis and Volga were provided by the cooperative Terre de Lin (Saint‐Pierre‐Le‐Viger, France); the cultivars Gisa, Primus, and Raba0189 were provided by the Leibniz Institute of Plant Genetics and Crop Plant Research (IPK, Gatersleben, Germany), for which herbarium sheets are available at the Genebank Information System of the institute and via the European Search Catalogue for Plant Genetic Resources (EURISCO); for *L. sufrutticosum*, a voucher is available at CSIC‐Real Jardín Botánico (MA, Madrid, Spain); W77 and *L. tenue* are part of a private (Gutaker et al., 2019) and live (El Castillejo Botanical Garden) collections, respectively. For *L. usitatissimum* cultivars, coordinates reflect the centroids of the country of origin.

^c^ Populations used for genome skimming are marked with the name of the individual used. These names were also used to mark the contigs deposited in GenBank (Appendix 2). A dash means that the population was not used for genome skimming.

## APPENDIX 2 Characteristics of 50 microsatellite loci tested in *Linum bienne*.

**Table:**

Locus	Contig	Chromosome^a^	Repeat motif	Forward primer	Reverse primer	*T*_a_ (°C)	Product size^b^	GenBank accession no.
1	chr1_L46_NODE_14803	chr1	(AGG)_3_	CTGTGAAGAGCAAGCTGACG	CGTTGAAAGTCTGACCGGTC	X	201	MN450480
2	chr1_L46_NODE_28525	chr1	(AGC)_3_	CTACCTCTCTCCGCCATAGC	TCGTCCTTCTCCATCGTCAC	56	240	MN450481
3	chr1_L68_NODE_33844	chr1	(ATC)_4_	TGGTGGACTGAGTTTCGGAG	TTATCGGCGCGTTGATGTTC	X	231	MN450482
5	chr1_L80_NODE_42754	chr1	(AC)_4_	AGGAGCCTGAAAGTCCATGG	ACATGTGATGCAATCCCAGC	56	228	MN450484
9	chr3_L46_NODE_99688	chr3	(AG)_5_	ACTCAAGTGAACCGCCCTAG	CTAATCCATCGGGCGTTTCC	55	155	MN450488
11	chr4_L17_NODE_131243	chr4	(AG)_6_	ACCACAACTGCTGCTTCATG	CTAAGTTGCACCGTGACCAC	X	221	MN450490
12	chr4_L46_NODE_135375	chr4	(ACC)_4_	GTGGTAGGAGACAGTACGGC	ATACCTGCTTTGTCTCCCGG	56	179	MN450491
15	chr4_L58_NODE_93648	chr4	(ACC)_5_	TTAGGTGGTTGTTGTTGCCC	CCTCGTCCCTCTAACCATCG	56	167	MN450494
16	chr5_L58_NODE_21665	chr5	(CCG)_4_	ACTCACCGTCACTGGGAATG	CGTCTCCAGCAGCAGATTTC	56	170	MN450495
17	chr5_L64_NODE_89794	chr5	(AAG)_5_	AGTGGGAGAGGGTTTGGTTG	AATGTGATTACTGGCGAGCG	57	207	MN450496
18	chr6_L46_NODE_63386	chr6	(AC)_9_	GGTTCAACGCCTCCAAGTTC	TCGGATGTGGCTTGAAACTG	56	128	MN450497
19	chr6_L46_NODE_7229	chr6	(AC)_4_	GAGCCTGACGATCTCTAGCC	CCACGAAGAAGCCAATGGTC	X	162	MN450498
20	chr6_L46_NODE_95299	chr6	(AGG)_4_	GCCGTACAGAACATCGTCAC	GTTGCCTCCCTCGAAATCTG	56	244	MN450499
22	chr7_L46_NODE_17919	chr7	(AGC)_5_	ACTCTACCGATCACAGACGC	ATGTGGGTGACTGATCCGAG	56	297	MN450501
23	chr7_L46_NODE_21601	chr7	(AAC)_3_	ACAGGGCGAATCTACAGACG	GCGTGTCGAGTGAACAAGAG	56	256	MN450502
24	chr7_L46_NODE_88119	chr7	(AAG)_3_	TTTCAGCTTCTTCTTCCCGC	GGAAACCGTGGGCTAATTCG	56	254	MN450503
25	chr8_L01_NODE_40486	chr8	(AAG)_5_	TCAAACACCATCTCCTCCGG	TGTGTCACGGCAATTCAAGC	56	160	MN450504
26	chr8_L46_NODE_63386	chr8	(AC)_9_	GGTTCAACGCCTCCAAGTTC	TCGGATGTGGCTTGAAACTG	56	128	MN450505
27	chr8_L46_NODE_7229	chr8	(AC)_4_	GAGCCTGACGATCTCTAGCC	CCACGAAGAAGCCAATGGTC	X	162	MN450506
28	chr8_L46_NODE_76521	chr8	(CCG)_3_	GATCCGGAGCTCAGACCATC	CTTCGGAATCACGGCTGTTG	56	199	MN450507
29	chr8_L46_NODE_95299	chr8	(AGG)_4_	GCCGTACAGAACATCGTCAC	GTTGCCTCCCTCGAAATCTG	56	244	MN450508
30	chr8_L68_NODE_29390	chr8	(AC)_3_	GTTACCATCCGCCTTCTTCG	GGCGTTTGGAAGAATGAGGG	56	217	MN450509
31	chr9_L01_NODE_43164	chr9	(AAG)_3_	ATCACCTCCTCCGCTCTTAC	ACGTGTTGTTGAAGCTGCTC	56	216	MN450510
32	chr9_L46_NODE_17919	chr9	(AGC)_5_	ACTCTACCGATCACAGACGC	ATGTGGGTGACTGATCCGAG	56	297	MN450511
33	chr9_L46_NODE_21601	chr9	(AAC)_3_	ACAGGGCGAATCTACAGACG	GCGTGTCGAGTGAACAAGAG	56	256	MN450512
34	chr9_L58_NODE_3456	chr9	(CCG)_4_	GCATGGCAGAAGAGTGATCG	CATCAGCAGTTCCACGTCAC	56	168	MN450513
36	chr9_L80_NODE_25256	chr9	(AG)_4_	TCGTCAGTTGAGCATTCGTG	CTCGCCACTTCTTTCGACAC	55	166	MN450515
37	chr10_L01_NODE_15945	chr10	(ATC)_3_	TCCACGTCATCACCTTCGTC	CGCAGTCAACTTTCGTACGG	56	183	MN450516
38	chr10_L64_NODE_9362	chr10	(AAC)_3_	AAGCACGCTGTTGTTTCTCC	AGGGTTGAAGAAGGAGCAGG	X	250	MN450517
45	chr12_L64_NODE_114187	chr12	(AG)_7_	AGCTCTTGAAGACGGCAAAC	GATCAACGGCGAATGACTGG	55	184	MN450524
46	chr13_L64_NODE_53800	chr13	(AG)_7_	TCAGTTCCTCCCACATCTCG	TTAGAGCATCCCAAGCCTCC	56	282	MN450525
47	chr13_L80_NODE_99677	chr13	(ACG)_6_	TGCCCAGGATGATGTGTAGC	CAAAGGCTTGCCAAATTGCC	56	155	MN450526
49	chr14_L01_NODE_48466	chr14	(AAC)_3_	AGGAACCTCAATACGCCGAG	GCTGCCTTGACGATTTCTCC	56	178	MN450528
50	chr15_L01_NODE_40627	chr15	(AG)_4_	GAAAGCGGTGACAGAGATGC	CCCATCACCCATCTCCCTTC	56	163	MN450529
ssr1.4	chr1_L68_NODE_46821	chr1	(AGC)_5_	CGAGCTCCGTTATCTCCGAG	ACGAATCTGAAATGGCGCTG	56	129	MN450483
ssr10.1	chr10_L68_NODE_100690	chr10	(AG)_5_	TCTACAATGGCGACTCAGGG	CGAATCGGTCAGCGGAATTG	56	120	MN450518
ssr11.1	chr11_L46_NODE_43040	chr11	(AAC)_5_	CTTCATCTCCGCTTGTTCCG	CATTGGCTGGGCAAGTATGG	56	185	MN450519
ssr11.2	chr11_L64_NODE_100592	chr11	(AAC)_4_	TGTGCGCAATATGGGTTACG	ACCCACCATCCTTTCTCCAC	56	243	MN450520
ssr11.4	chr11_L64_NODE_14339	chr11	(AG)_4_	AAACCAACATCCCACTTGCG	TTCCAACTGAAAGACGCTCG	56	300	MN450521
ssr12.3	chr12_L46_NODE_28661	chr12	(AAG)_5_	GGCCACGAATTCCCTCATTC	TGGGAAGAACAGTACGGTCC	56	225	MN450523
ssr12.4	chr12_L01_NODE_38654	chr12	(AG)_5_	CTACCCTTCTCAGCTCTGCC	TTGTGTGCACTTCAAAGCCC	56	173	MN450522
ssr14.3	chr14_L01_NODE_12417	chr14	(ACT)_4_	ACATTCGCAACTGTATCGCC	GCGTTTAGGTGGTGGAAAGG	56	279	MN450527
ssr2.1	chr2_L46_NODE_13038	chr2	(AGG)_4_	AAAGAAATGCAGAGCGGGAG	GCGTCATTTACTCAGTGGCC	56	211	MN450485
ssr2.2	chr2_L49_NODE_29522	chr2	(AG)_5_	CCGTTGCTCTTCCACCAAAG	CATCTTCACCGTTCAGCTCG	56	277	MN450486
ssr3.2	chr3_L68_NODE_6280	chr3	(AT)_8_	GTCTGCATTGCGATCAGAGG	GATAGGTGCCTTGTTCTGCG	56	157	MN450489
ssr3.4	chr3_L46_NODE_33336	chr3	(AT)_8_	CAGATTCAACCGTTGCTCCC	TTGCCTGTTTCCAACGAGAC	56	227	MN450487
ssr4.2	chr4_L49_NODE_25476	chr4	(ATC)_5_	TCGTCCTTGATCCTTCCAGC	AAGACCCTCAACTCCAACCC	56	198	MN450493
ssr4.3	chr4_L46_NODE_22236	chr4	(AAG)_5_	ATAGCTGCCAACTTGACTGC	TTTCCTAGGACCAGCGACTG	56	129	MN450492
ssr6.1	chr6_L64_NODE_173634	chr6	(AG)_6_	TTACACGAGGGATTGCAAGC	ACTAGTGAGTCTGCAGTGCC	56	161	MN450500
ssr9.3	chr9_L68_NODE_2216	chr9	(AC)_4_	TACGCCAAACACAAGCATCC	CAACCCAACCATACCAACCG	56	185	MN450514

Note

*T*_a_ = optimized annealing temperature for each primer pair; X = unsuccessful amplification.

^a^ The loci were obtained via genome skimming using the *L. usitatissimum* genome as reference; therefore, it was possible to identify a putative chromosome for each locus.

^b^ The product sizes reported here are based on MSATCOMMANDER output, although the sizes were double‐checked by looking at the agarose gels of the PCR products for all loci, where a ladder was added to assist the estimation of the products’ approximate size.
