# Supplementary material for: Microsatellite marker development in the crop wild relative Linum bienne using genome skimming
Source: Appl Plant Sci. 2020 May 26;8(5):e11349. doi: 10.1002/aps3.11349 (PMC7249271; doi:10.1002/aps3.11349)
Supplement: Supplementary file 1 — APPENDIX S1. Script for de novo genome assembly using six Linum bienne individuals of different geographical origin, subsequent microsatellite loci mining and primer design, and in silico genotyping. [file APS3-8-e11349-s001.docx]

**APPENDIX S1.** Script for de novo genome assembly using six *Linum bienne* individuals of different geographical origin, subsequent microsatellite loci mining and primer design, and in silico genotyping.

**Notes:**

1. The symbol # is used for commenting.
2. At the beginning of each part (4 parts in total) it is indicated whether the code is to be executed in R or in bash.

**PART 1. QUALITY CHECKS, ASSEMBLY DE NOVO, AND SCAFFOLDS MAPPING TO *LINUM USITATISSIMUM* REFERENCE NUCLEAR GENOME**

This part is executed in bash.

A de novo assembly was executed for six *L. bienne* samples of different origin. Scaffolds were mapped to the *L. usitatissimum* reference genome and what mapped to unique genomic regions was retained for downstream analysis (part 2).

**Code**

# Check quality of raw reads in Fastqc

fastqc /directory/raw/reads/*.fq.gz -o /directory/raw/reads -noextract

# With loop, trim raw reads in trimmomatic, check quality of trimmed reads with fastqc and produce report comparing trimmed VS raw reads with multiqc

## adapterIL.fa is file containing adapters specific to sequencing platform

for f1 in /directory/raw/reads/*F.fq.gz

do

echo "working with file $f1"

dir="/directory/raw/reads"

f2=${f1%%F.fq.gz}"R.fq.gz"

f1p=${dir}/$(basename -s .fq.gz $f1)_P.filtered.fq.gz

f1u=${dir}/$(basename -s .fq.gz $f1)_U.filtered.fq.gz

f2p=${dir}/$(basename -s .fq.gz $f2)_P.filtered.fq.gz

f2u=${dir}/$(basename -s .fq.gz $f2)_U.filtered.fq.gz

trimmomatic PE -phred33 -trimlog ${dir}/TrimLog $f1 $f2 $f1p $f1u $f2p $f2u ILLUMINACLIP:/directory/adapters/file/adapterIL.fa:2:30:10 AVGQUAL:20 MINLEN:40

fastqc /directory/raw/reads/*P.filtered.fq.gz -o /directory/raw/reads -noextract

multiqc -ip /directory/raw/reads/*_fastqc.zip

done

# Do assembly with trimmed, paired-end reads in spades

module load apps/spades/3.13

for fq1 in `ls /directory/raw/reads/*_F_P.filtered.fq.gz`

do

dir="/directory/raw/reads"

base=$(basename $fq1 "_F_P.filtered.fq.gz")

echo "basename is $base"

fq1=${dir}/${base}_F_P.filtered.fq.gz

fq2=${dir}/${base}_R_P.filtered.fq.gz

out=${dir}/${base}_out

spades.py -k 21,33,55,77 --careful -1 $fq1 -2 $fq2 -o $out

done

# Map scaffolds generated with spades to Linum usitatissimum nuclear reference genome using bwa. Only scaffolds that map to nuclear genome are kept.

## The genome is divided into chromosomes, so the same process has to be looped through chromosomes.

## Get fasta files for chromosomes at https://www.ncbi.nlm.nih.gov/genome/?term=linum%20usitatissimum&utm_source=gquery&utm_medium=search.

## Save each chromosome sequence in a folder named after the given chromosome ("Chr1", "Chr2", ..., "Chr15")

## Loop through folders to map all scaffolds every time to a different chromosome.

cd /directory/raw/reads/bwaFolder/

export PATH="$PATH:/.../bcftools-1.9" ###maybe not necessary, depends on installation

export PATH="$PATH:/.../samtools-1.9"

export PATH="$PATH:/.../htslib-1.9"

source ~/.profile

for dir in /directory/raw/reads/bwaFolder/*/

do

dir=${dir%*/}

echo ${dir##*/}

export dir

pushd $dir

pwd

for f1 in scaffolds*fasta

do

base=$(basename $f1 ".fasta")

chr=$(basename $dir)

cnt=${base}.fasta

sam=${base}.sam

bam=${base}.bam

srt=${base}.sorted.bam

mpd=${base}.mapped.bam

mpds=${base}.mapped.sorted.bam

echo "base name is $base"

bwa mem ${chr}.fasta $cnt > $sam

samtools view -bS $sam > $bam

samtools sort $bam -o $srt

samtools index $srt

samtools view -b -F 4 $srt > $mpd ###-F 4 excludes all scaffolds that did NOT map

samtools sort $mpd -o $mpds

samtools index $mpds

done

popd

done

## Check that I retained only scaffolds that UNIQUELY mapped (mapped to only one region in the genome)

## 260 stands for 256 (mapped to multiple regions) + 4 (unmapped) and -f looks for things corresponding to those criteria, so I should get zero if all I have left is uniquely mapped

samtools view -f 260 name.mapped.sorted.bam | wc -l

## From here, I cannot see if there are scaffold that have mapped to multiple reference chromosomes, since reference chromosomes were kept separated. I can check for this later in R and remove them if there are any.

## Use bam files to get fasta sequences of scaffolds that mapped in each chromosome folder

for dir in /directory/raw/reads/bwaFolder/*/

do

dir=${dir%*/}

echo ${dir##*/}

export dir

pushd $dir

pwd

for f1 in scaffolds*.mapped.sorted.bam

do

base=$(basename $f1 ".mapped.sorted.bam")

in=${base}.mapped.sorted.bam

out=${base}.mapped.sorted.fasta

echo "base name is $base"

samtools fasta $in > $out

done

popd

done

## Add chromosome number and sample number to multi fasta headers.

## That is useful because I am able to track from where each sequence came from when passing the fasta files to MSATCOMMANDER and afterwards.

for dir in /directory/raw/reads/bwaFolder/*/

do

dir=${dir%*/}

echo ${dir##*/}

export dir

pushd $dir

pwd

for f1 in scaffolds_*.mapped.sorted.fasta

do

base0=$(basename $f1 ".mapped.sorted.fasta")

rmv="scaffolds_"

base=${base0//$rmv/}

echo "base name is $base"

chr=$(basename $dir)

hdr0=${chr}_${base}_

hdr=">$hdr0"

echo "header is $hdr"

nmd=${rmv}${base}.${chr}.mapped.sorted.fasta

bioawk -v hdr1=$hdr -c fastx '{ print hdr1 $name "\n" $seq }' $f1 > $nmd

done

popd

done

## Merge 15 fasta files with uniquely mapped scaffolds for each chromosome into one.

## Then split it into 4 smaller chunks with fasta splitter (http://kirill-kryukov.com/study/tools/fasta-splitter/) so that you can run MSATCOMMANDER on different computers at the same time.

### The four chunks so generated are used as they are to feed MSATCOMMANDER which scans for microsatellite loci and designs primers

### MSATCOMMANDER does not have the option of parallel processing and it must be run locally, so creating chunks is a way to speed up things if you have access to multiple computers

cat /directory/raw/reads/bwaFolder/*mapped.sorted.fasta > multisample.mapped.sorted.fa

fasta-splitter.pl --n-parts 4 multisample.mapped.sorted.fa --out-dir /directory/raw/reads/bwaFolder/

## MSATCOMMANDER needs to be downloaded at <https://code.google.com/archive/p/msatcommander/> (Program works better on Mac)

**PART 2. FILTERING OF LOCI AND PRIMERS BASED ON LOCI AND PRIMERS FEATURES**

This part is executed in R.

First, I exclude scaffolds that might have mapped to multiple chromosomes, then I merge different MSATCOMMANDER outputs (ssr loci + primers relative to loci), then I filter primers based on their qualities.

**Code**

#Libraries

Library(tidyverse)

# Set working directory

setwd("/dir/with/MSATCOMMANDER/output/")

# Merge and tidy MICROSATELLITE LOCI files

## We had 4 fasta chunks that we fed to msatcommander so we have 4 outputs that we have to merge, both for microsatellite loci and relative primers

s1 <- read.csv("msatcommander.microsatellites.1.csv")

s2 <- read.csv("msatcommander.microsatellites.2.csv")

s3 <- read.csv("msatcommander.microsatellites.3.csv")

s4 <- read.csv("msatcommander.microsatellites.4.csv")

str(s1)

## Merge name and id columns into name_id column.

### Column name contains reference chromosome number, sample number, scaffold number

### Column id contains number assigned by msatcommander to given locus in given scaffold

### This way I can uniquely identify different loci on same scaffold

s1$name_id <- paste(s1$name, s1$id, sep="_")

str(s1)

s2$name_id <- paste(s2$name, s2$id, sep="_")

s3$name_id <- paste(s3$name, s3$id, sep="_")

s4$name_id <- paste(s4$name, s4$id, sep="_")

## Just add column named "fasta" that tells us which fasta chunk the locus was found in

## Then bind dataframes for each chunk into one and save the file

## NB:

#### When mapping the scaffolds against the reference genome with bwa, we filtered out what mapped to multiple locations within each chromosome, as L. usitatissimum reference genome is divided into chromosomes.

#### We could not know though, if the same scaffold mapped to multiple locations distributed across different chromosomes.

#### Duplicates we have at this step, are then given by scaffolds that mapped to multiple chromosomes and we want to eliminate loci falling in these scaffolds, so we eliminate duplicates.

s1["fasta"] <- "1"

str(s1)

s2["fasta"] <- "2"

s3["fasta"] <- "3"

s4["fasta"] <- "4"

ssr <- rbind(s1, s2, s3, s4)

str(ssr)

ssr$qseqid_motif <- as.factor(paste(ssr$name, ssr$motif, sep="_"))

ssr <- ssr[!duplicated(as.character(ssr$qseqid_motif)), ]

str(ssr)

write.csv(ssr, "msatcommander.microsatellites.nameid.all.csv")

# Merge and tidy PRIMERS files

p1 <- read.csv("msatcommander.primers.1.csv")

p2 <- read.csv("msatcommander.primers.2.csv")

p3 <- read.csv("msatcommander.primers.3.csv")

p4 <- read.csv("msatcommander.primers.4.csv")

str(p1)

## Same as with loci: name and id columns are merged for unique id

## NB: msatcommander assigns same id number to ssr locus and relative primer pair

p1$name_id <- paste(p1$name, p1$msats_id, sep="_")

str(p1)

p2$name_id <- paste(p2$name, p2$msats_id, sep="_")

p3$name_id <- paste(p3$name, p3$msats_id, sep="_")

p4$name_id <- paste(p4$name, p4$msats_id, sep="_")

## For same locus though, there might be multiple primer pairs options.

## I delete all rows corresponding to potentially duplicated primers.

which(p1$name == "Potentially duplicated primers:")

p1.nd <- droplevels(p1[1:28990,])

which(p1.nd$name == "Potentially duplicated primers:") #Should give zero or null now

which(p2$name == "Potentially duplicated primers:")

p2.nd <- droplevels(p2[1:43907,])

which(p2.nd$name == "Potentially duplicated primers:")

which(p3$name == "Potentially duplicated primers:")

p3.nd <- droplevels(p3[1:29786,])

which(p3.nd$name == "Potentially duplicated primers:")

which(p4$name == "Potentially duplicated primers:")

p4.nd <- droplevels(p4[1:21828,])

which(p4.nd$name == "Potentially duplicated primers:")

## As for loci, I add column to know from which fasta chunk primers come from, then merge all dataframes into one.

## I can save merged cleaned file if I want.

p1.nd["fasta"] <- "1"

str(p1.nd)

p2.nd["fasta"] <- "2"

p3.nd["fasta"] <- "3"

p4.nd["fasta"] <- "4"

prm <- rbind(p1.nd, p2.nd, p3.nd, p4.nd)

str(prm)

#write.csv(prm, "msatcommander.primers.nameid.all.csv")

# Merging MICROSATELLITE LOCI and relative PRIMERS data frames

## Merging is done by name_id column because it uniquely identifies ssr loci and relative primers

## I can save merged file if I want

mr <- merge(x = ssr, y = prm, by = "name_id")

str(mr)

# Filter LOCI and PRIMERS based on PRIMERS qualities

## Create new columns used later on for distinguishing loci and calculate filtering parameters values by splitting old columns that contain multiple info

mrc <- mr %>%

separate(col = left, into = c("left_start","left_length"), sep = ",", remove = TRUE) %>%

separate(col = right, into = c("right_start","right_length"), sep = ",", remove = TRUE) %>%

separate(col = name_id, into = c("chr_samp", "name_wo"), sep = "_NODE", remove = FALSE) %>%

mutate(

dtm = abs(left_tm - right_tm, #difference in melting temperature between f and r primers

left_pos = as.numeric(left_start) + as.numeric(left_length), #position for left primer end

right_pos = as.numeric(right_start) - as.numeric(right_length), #position for right primer end

ssr_left = start - left_pos, #distance from start ssr to end left primer

ssr_right = end - right_pos, #distance from start ssr to end right primer

chr = as.factor(as.character(chr)),

samp = as.factor(as.character(samp))

)

str(mrc)

## Subset by primers features

mrcs <- mrc %>% subset(pair_penalty < 1.7 & left_penalty < 0.8 & right_penalty < 0.8) %>%

subset(dtm < 2) %>%

subset(count >= 5) %>%

subset(ssr_left > 20 & ssr_right < -20) %>%

subset(pair_product_size > 89 & pair_product_size < 301) %>%

as.data.frame() %>%

droplevels()

str(mrcs)

### Just to have an idea of how many loci per chromosome and sample I found

summary <- as.data.frame(summarise(group_by(mrcs,chr,samp), count=n()))

View(summary)

## Later on, I'll need a bed file with filtered ssrs to use as database for blasting all ssrs to search for eventual polymorphisms.

mrcs$start.bed <- (mrcs$start - 40)

mrcs$end.bed <- (mrcs$start + 40)

str(mrcs)

write.csv(mrcs, "merged.subset.csv")

myvars <- c("name.x", "start.bed", "end.bed", "id")

bed <- mrcs[ ,myvars]

str(bed)

bed$id <- paste0("ID=", bed$id)

bed.nh <- bed

names(bed.nh) <- NULL

write.table(bed.nh, "merged.subset.bed", sep="\t", quote=FALSE, row.names=FALSE)

**PART 3. BLAST ALL SCAFFOLDS AGAINST FILTERED LOCI SUBSET**

This part is executed in bash.

The bed file containing the loci + primers subset obtained in above section is used as database to search against. Query sequences are all uniquely mapped scaffolds.

This way, we can find if there are scaffolds’ regions containing SSR loci from *L. bienne* individual A that match to scaffolds from *L. bienne* individual B and whether the matching ssr loci differ in length between the two individuals.

**Code**

## set directory

cd /dir/with/MSATCOMMANDER/output/

## get filtered loci fasta and make search database

bedtools getfasta -fo merged.subset.bed.fasta -fi multisample.mapped.sorted.fasta -bed merged.subset.bed

makeblastdb -in merged.subset.bed.fasta -dbtype nucl

## run blast search

### for details about file format (-outfmt) options see http://www.metagenomics.wiki/tools/blast/blastn-output-format-6

### the various options used here are necessary for filtering loci based on similarity of sequences and polymorphisms at ssr locus

blastn -db merged.subset.bed.fasta -query multisample.mapped.sorted.fasta -out multisample.mapped.sorted.blast60.tab -perc_identity 60 -outfmt "6 qseqid sseqid pident qstart qend qlen qseq sseq length mismatch gaps gapopen evalue bitscore"

**PART 4. IN SILICO GENOTYPING BASED ON BLAST SEARCH**

This part is executed in R.

All datasets (ssr, filtered ssr+primers, and blast output) are merged together to double check for discrepancies, then genotyping is executed by counting the maximum number of consecutive gaps (“-“ symbol) and checking that it is a multiple of the base motif size. Final output (csv) needs to be checked manually.

**Code**

## Libraries

library(tidyverse)

## Set working directory

setwd("/dir/with/MSATCOMMANDER/output/")

## Import various data files needed and format them so that they match

### Import file with all ssr saved in PART 1

ssr <- read.csv("msatcommander.microsatellites.nameid.all.csv")

str(ssr)

ssr$qseqid_motif <- as.factor(paste(ssr$name, ssr$motif, sep="_"))

### Import blastn output saved in PART 3

gntp <- read.table("multisample.mapped.sorted.blast60.tab")

str(gntp)

nrow(gntp)

hdrs <- c("qseqid","sseqid","pident","qstart", "qend", "qlen_tot","qseq","sseq","al_length","mismatch","gaps", "gapopen", "evalue","bitscore") #blastn output does not have headers, so I have to add them

colnames(gntp) <- hdrs

str(gntp)

min(gntp$mismatch) #just to have an idea of mismatches and gaps

max(gntp$mismatch)

min(gntp$gaps)

max(gntp$gaps)

### Import file with filtered ssr and primers also saved in PART 1

mrcs <- read.csv("merged.subset.csv")

str(mrcs)

mrcs$sseqid1 <- paste(mrcs$node, mrcs$start.bed, sep=":") #to create column with sseqid column which corresponds to sseqid in blastn dataframe

mrcs$sseqid2 <- paste(mrcs$sseqid1, mrcs$end.bed, sep="-")

mrcs$sseqid <- paste(mrcs$chr, mrcs$samp, mrcs$sseqid2, sep="_")

nrow(mrcs[!duplicated(mrcs$sseqid), ]) #just doublecheck that there are no duplicates in the sseqid column this way

nrow(mrcs)

## Merge blastn and msatcommander dataframes and delete unnecessary columns

mrg.p <- merge(x = gntp, y = mrcs, by = "sseqid") #merge keeps only rows that match, non-matches are discarded

str(mrg.p)

### Change order of column (just handy)

mrg.ps <- mrg.p %>%

select(chr, samp, node, name.x, id, name_id, qseqid, sseqid, everything()) %>%

select(-X, -name_wo, -name.y, -records_id.y, -records_id.x, -fasta.y, -sseqid1, -sseqid2)

colnames(mrg.ps)[4] <- "name.ss"

### Separate seq name for db sequence and query sequence using "_" as separator and delete unecessary columns and change new columns from character to factor

mrg.2 <- separate(mrg.ps, col = name.ss, into = c("chr.ss", "smp.ss", "del1.ss", "node.ss", "del2.ss", "length.ss", "del3.ss", "cov.ss"), sep = "_", remove = FALSE)

mrg.3 <- separate(mrg.2, col = qseqid, into = c("chr.qs", "smp.qs", "del1.qs", "node.qs", "del2.qs", "length.qs", "del3.qs", "cov.qs"), sep = "_", remove = FALSE)

cols <- c("chr.ss", "smp.ss", "node.ss", "length.ss", "cov.ss", "chr.qs", "smp.qs", "node.qs", "length.qs", "cov.qs")

mrg.3 <- mrg.3 %>%

select(-starts_with("del")) %>%

mutate_each_(funs(factor(.)), cols) %>%

mutate(qseqid_motif = as.factor(paste(mrg.3$qseqid, mrg.3$motif, sep="_")))

str(mrg.3)

## Merge with ssr data keeping all rows from mrg.3 to add query sequence info

mrg.4 <- merge(x = ssr, y = mrg.3, by = "qseqid_motif", all.y = TRUE)

str(mrg.4)

mrg.4$q_name_id <- paste(mrg.4$name, mrg.4$id.x, sep="-")

mrg.5 <- mrg.4 %>%

group_by(name_id.y) %>%

subset(chr.ss == chr.qs) %>% # make sure chromosome is same for query and db seqs (by row)

select(-X, -preceding.x, -following.x, -preceding.y, -following.y, -chr_samp.x, -chr_samp.y, -msats_id, -primer, -chr.x, -samp.x, -node.x, -chr.y, -samp.y, -node.y) %>%

ungroup %>%

rename(id.qs = id.x,

motif.qs = motif.x,

start.node.qs = start.x,

end.node.qs = end.x,

count.qs = count.x,

name_id.qs = name_id.x,

id.ss = id.y,

name_id.ss = name_id.y,

motif.ss = motif.y,

start.node.ss = start.y,

end.node.ss = end.y,

count.ss = count.y) %>%

as.data.frame()

str(mrg.5)

nrow(mrg.4)

nrow(mrg.5)

## Add columns for max number of consecutive gaps in search sequence, number of max consecutive gaps in query sequence, and motif size

mrg.5$gap.max.ss <- sapply(strsplit(as.character(mrg.5$sseq), ""), function(x) {

inds = rle(x)

max(inds$lengths[inds$values == "-"])

})

mrg.5$gap.max.qs <- sapply(strsplit(as.character(mrg.5$qseq), ""), function(x) {

inds = rle(x)

max(inds$lengths[inds$values == "-"])

})

mrg.5$motif.size <- nchar(as.character(mrg.5$motif.ss), type = "chars", allowNA = FALSE, keepNA = NA)

## Genotyping in silico based on number of gaps and mismatches

mrg.6 <- mrg.5 %>%

group_by(name_id.ss) %>%

filter(any(gaps >=2)) %>% #at least one entry per group has two or more gaps

filter(any(gap.max.ss == gap.max.ss[(gap.max.ss %% motif.size) == 0] | gap.max.qs == gap.max.qs[(gap.max.qs %% motif.size) == 0])) %>% #keep only rows for which the maximum number of gaps in query or search sequence is a multiple of ssr motif length

ungroup() %>%

filter(mismatch < 5) %>% #keep only rows where mismatch between search and query sequence is smaller than 5 (less than 5 snps)

filter(count.ss >= 4) %>% #motif repeated at least 4 times in search sequence

droplevels() %>%

as.data.frame()

View(as.data.frame(summarise(group_by(mrg.6, chr.ss, smp.ss), count = n()))) #to check if enough loci were retained

## Create groups that should be representative of same locus

### Then check manually if accurate by opening saved csv

mrg.7 <- mrg.6 %>%

group_by(chr.ss, motif.ss, name_id.ss) %>%

mutate(count = n()) %>%

ungroup() %>%

mutate(locus.group = paste0(chr.ss, "_", motif.ss, "_", count)) %>%

group_by(locus.group) %>%

distinct(name_id.qs, .keep_all = TRUE) %>%

as.data.frame()

str(mrg.7)

write.csv(mrg.7, "selected.loci.raw.csv")
