# Supplementary material for: Microsatellite marker development in the crop wild relative Linum bienne using genome skimming
Source: Appl Plant Sci. 2020 May 26;8(5):e11349. doi: 10.1002/aps3.11349 (PMC7249271; doi:10.1002/aps3.11349)

DOI

APPENDIX S2. Index of association (IA) for 16 polymorphic loci included in this study. Color gradient represents IA values and numbers represent P values for the significance of the association (association significant when $P < 0.05$). IA is represented for each *Linum bienne* population.

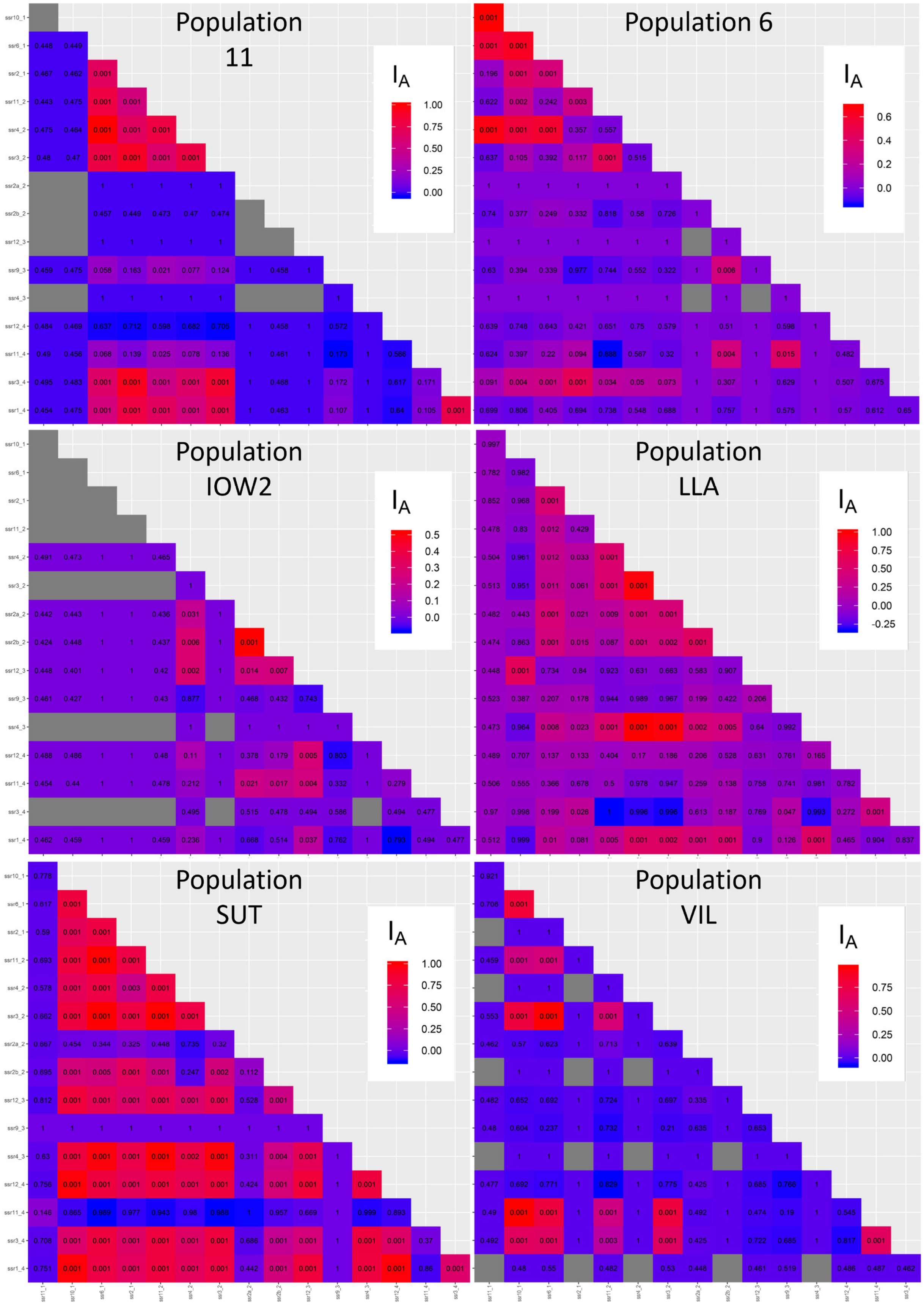

Supplement: Supplementary file 2 — APPENDIX S2. Index of association for 16 polymorphic loci included in this study. [file APS3-8-e11349-s002.pdf]
